# Modeling-Guided Amendments Lead to Enhanced Biodegradation in Soil

**DOI:** 10.1128/msystems.00169-22

**Published:** 2022-08-01

**Authors:** Kusum Dhakar, Raphy Zarecki, Shlomit Medina, Hamam Ziadna, Karam Igbaria, Ran Lati, Zeev Ronen, Hanan Eizenberg, Shiri Freilich

**Affiliations:** a Newe Ya'ar Research Center, Agricultural Research Organization, Ramat Yishay, Israel; b Department of Environmental Hydrology & Microbiology, Zuckerberg Institute for Water Research, Jacob Blaustein Institutes for Desert Research, Ben-Gurion University of the Negev, Midreshet Ben-Gurion, Israel; Lawrence Berkeley National Laboratory

**Keywords:** herbicides, biostimulation, metabolic modeling, microbial degradation

## Abstract

Extensive use of agrochemicals is emerging as a serious environmental issue coming at the cost of the pollution of soil and water resources. Bioremediation techniques such as biostimulation are promising strategies used to remove pollutants from agricultural soils by supporting the indigenous microbial degraders. Though considered cost-effective and eco-friendly, the success rate of these strategies typically varies, and consequently, they are rarely integrated into commercial agricultural practices. In the current study, we applied metabolic-based community-modeling approaches for promoting realistic *in terra* solutions by simulation-based prioritization of alternative supplements as potential biostimulants, considering a collection of indigenous bacteria. Efficacy of biostimulants as enhancers of the indigenous degrader *Paenarthrobacter* was ranked through simulation and validated in pot experiments. A two-dimensional simulation matrix predicting the effect of different biostimulants on additional potential indigenous degraders (Pseudomonas, *Clostridium*, and *Geobacter*) was crossed with experimental observations. The overall ability of the models to predict the compounds that act as taxa-selective stimulants indicates that computational algorithms can guide the manipulation of the soil microbiome *in situ* and provides an additional step toward the educated design of biostimulation strategies.

**IMPORTANCE** Providing the food requirements of a growing population comes at the cost of intensive use of agrochemicals, including pesticides. Native microbial soil communities are considered key players in the degradation of such exogenous substances. Manipulating microbial activity toward an optimized outcome in efficient biodegradation processes conveys a promise of maintaining intensive yet sustainable agriculture. Efficient strategies for harnessing the native microbiome require the development of approaches for processing big genomic data. Here, we pursued metabolic modeling for promoting realistic *in terra* solutions by simulation-based prioritization of alternative supplements as potential biostimulants, considering a collection of indigenous bacteria. Our genomic-based predictions point at strategies for optimizing biodegradation by the native community. Developing a systematic, data-guided understanding of metabolite-driven targeted enhancement of selected microorganisms lays the foundation for the design of ecologically sound methods for optimizing microbiome functioning.

## INTRODUCTION

Providing the food requirements of a growing population comes at the cost of an intensive use of agrochemicals, including pesticides, and leads to persistent residues that consistently exert negative effects on the ecosystem (1–3). To date, soil contamination caused by pesticides and herbicides is considered among the top 10 environmental hazards, with limited solutions that support green and cost-effective soil detoxification processes. Eco-friendly solutions for the decontamination of cultivated soils are a major goal of agricultural research (4–6). Native microbial soil communities are viewed as key players in the degradation of exogenous substances (7, 8). The soil microbial community includes microorganisms that participate either directly or indirectly in degradation processes and that drive the fluxes of energy and mass conversion through various interactions (9). Manipulation of microbial activity toward an optimized outcome in terms of efficient biodegradation processes conveys a promise of maintaining intensive yet sustainable agriculture (10, 11).

Backed by the need to develop sustainable strategies for the cleanup of agricultural soil, bioremediation solutions (use of living organisms for removing contaminants from the environment) are increasingly sought (12–14). The two fundamental strategies in bioremediation, bioaugmentation (addition of cultured microorganisms to improve the degradation process in the environment) and biostimulation (modifying the environment to support the growth of degraders), have been shown successful in accelerating the removal of specific synthetic chemicals (pesticides, herbicides, etc.) in agricultural soils (15–17). Biostimulants such as organic and inorganic compounds, biochar, and crop residues have been tested in several studies for their effects on the rapid removal of contaminants (18–20). In some studies, carbohydrates were identified as biostimulants that promoted indigenous soil degraders to enhance the degradation rate (21).

Although these strategies are considered cost-effective and eco-friendly, their success rate typically varies, and consequently, they are rarely integrated into commercial agricultural practices (12). Environmental genomics conveys the promise of revolutionizing bioremediation processes. Traditionally, biostimulation practices are developed based on an exhaustive process of trial and error, screening a limited number of possible solutions. This can be overcome by implementing algorithms for processing genomic “big data” that will provide a new toolkit for a better understanding of complex biological systems (22–25). In recent years, metabolic modeling has been increasingly used for exploring and improving selected metabolic performances of microbial species (26, 27). Modeling is based on *in silico* representation of genomic data obtained from field samples, supporting the conductance of multiple simulations leading to organism- and community-level phenotyping and the subsequent development of metabolic engineering strategies for biostimulation (28–31). Simulations can be designed to screen for the stimulation potential of metabolites for particular catabolic pathways, microbial species, and combinations of both. For a specific degradation process, exploiting the exhaustive power of computational modeling platforms allows a fast screening and prioritization of thousands of bioremediation solutions.

In a recent series of research works, we provided pioneering evidence that genomic-based algorithms can serve in the development of biostimulation approaches of several herbicides, including atrazine (32–35). Atrazine is a widely used herbicide for maize, sorghum, and sugarcane as a control against broadleaf weeds (32, 36). Extensive use of atrazine and its persistence in the environment led to its detection in various environmental samples (37). Although it is an effective agrochemical in increasing crop productivity, its persistence results in pollution of soil, surface water, and groundwater (38). To enhance the rate of degradation of atrazine in contaminated soils, the combination of sequencing technologies and metabolic modeling was shown efficient for designing strategies that will optimize its degradation by indigenous soil bacteria under *in vitro* conditions (32, 33, 34). Here, we pursued metabolic modeling for promoting realistic *in terra* solutions by simulation-based prioritization of alternative supplements as potential biostimulants, considering a collection of indigenous bacteria. A two-dimensional simulation matrix was created to explore the potential impact of different carbon sources as biostimulants of different native degraders. The effects of different biostimulants on the performances of the microbial community and the relative abundance levels of individual species were validated in pot experiments.

## RESULTS

### Ranking potential biostimulants for an indigenous degrader of the herbicide atrazine.

In a previous study, an *Arthrobacter* species (NCBI accession MG554188) was identified as an efficient degrader of atrazine in soil taken from a commercial field in Newe-Ya’ar, Israel (32). The native species is highly similar (98% identity) to the well-studied atrazine degrader Paenarthrobacter aurescens TC1 (34, 39, 40). Despite clear evidence for atrazine degradation activity in general and in the specific soil in particular, the relative abundance of the *Arthrobacter* species was unaffected by application of atrazine at conventional rates in commercial agricultural practices (32). The nonsignificant change in the abundance of a key degrader could be related to a weak impact of a single factor (atrazine) in a complex multivariate system, such as soil with multiple physical and chemical properties. The use of organic supplements as enhancers of atrazine degradation in both *in vitro* and *in terra* systems (32, 41) indicates that atrazine degradation can be optimized beyond the response achieved by the inductive effect of atrazine *per se*. In order to generate a maximal stimulation effect, we aimed to construct a simulation system that allowed us to predict the outcomes of different additives on the rate of degradation in a community with multiple degraders.

**FIG 1:**
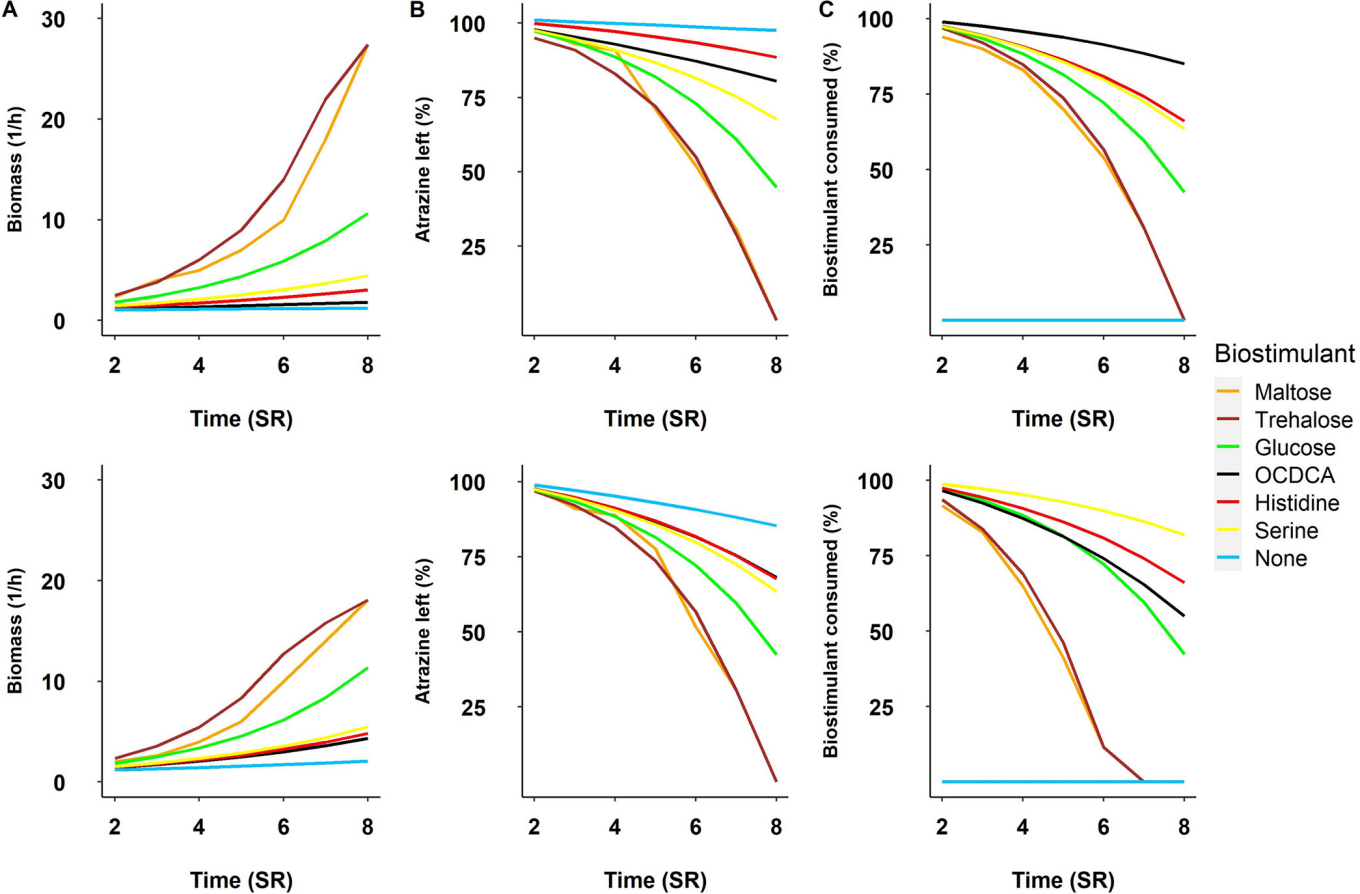
Simulations of metabolic performances of *P. aurescens* TC1 in minimal media supplemented with different potential biostimulants, in terms of growth (A), atrazine degradation (in millimoles per gram [dry weight]) (B), and consumption of degradation (in millimoles per gram [dry weight]) (C). The top and bottom series of panels represent supplementation with a standard amount of stimulant (50 mmol/g [dry weight]; top) or with fluxes normalized according to molecular carbon content (bottom). In the carbon-normalized simulations, the initial amount of maltose, trehalose, glucose, OCDCA, histidine, or serine was 25, 25, 50, 16, 50, or 100 mmol/g (dry weight), respectively. Time (SR) graphs show results of a times series with simulation rounds representing cycle-limited quantities of nutrient uptake from the environment (see Materials and Methods).

To this end, we applied dynamic simulations for testing the relative efficacies of all exchange metabolites (total of 109) of the metabolic model of P. aurescens TC1 as potential biostimulants of atrazine degradation (see Table S1 in the supplemental material). As expected from various laboratory and field studies (21, 32), carbohydrates including glucose were found to have a strong impact on the atrazine degradation. Though glucose is indeed an efficient enhancer of degradation, the simulation points to disaccharides as the most efficient stimulants (see Fig. S1). The stimulation activity of the top biostimulants trehalose and maltose (C_12_), in comparison to glucose (C_6_), the carbon-rich compound octadecanoate (OCDCA) (C18) and two amino acids that are weak enhancers of degradation in comparison to sugars (Fig. 1; see also Fig. S1). The amino acids include serine, an efficient biostimulant among amino acids, and histidine, whose simulative effect depends on the presence of atrazine (and is more significant in its absence) (34). OCDCA, despite being a carbon-rich compound, is predicted to be a nonefficient enhancer, indicating that the number of carbons by itself is insufficient for predicting simulation efficiency (Fig. 1A, B, and C). To verify that the efficiency of the disaccharides in comparison to glucose did not solely reflect their higher carbon content, simulations were carried while normalizing input fluxes according to carbon content (Fig. 1, bottom) rather than molar values (Fig. 1, top). In accordance with previous studies, the comparison indicated that the simulations reflected a complexity that goes beyond carbon content and cannot be predicted based solely on biochemical characteristic (34). Simulations were also carried out with normalized nitrogen content and these resulted in overall conserved predictions (see Fig. S3).

10.1128/msystems.00169-22.1 FIG S1 We simulated growth in 109 different medium combinations, each supplementing the atrazine-containing minimal mineral medium with a single exchange metabolite. Of 109 potential supplements, a total of 75 metabolites were retained after eliminating nonpractical additives, such as dipeptides and toxic substances, from the initial set of exchange metabolites. Blue, carbon source; red, both carbon and nitrogen source; green, nitrogen source; black, no supplement. Rows represent rounds 1 to 15. The atrazine amount is represented from 100 to 0 as purple to yellow, respectively. NAG, *N*-acetyl-d-glucosamine; KDG, 2-keto-3-deoxy-d-gluconate; GP, glycerol-3-phosphate; MA, myristic acid; AE, aminoethanol; PA, l-phenylalanine; DA, 5′-deoxyadenosine; LMSO, l-methionine-*S*-oxide; MPSA, morpholinopropane sulfonic acid; MRO, l-methionine *R*-oxide; CO, carbon monoxide; PP, 2-propanamine. Download FIG S1, DOCX file, 0.2 MB. Copyright © 2022 Dhakar et al. 2022 Dhakar et al. https://creativecommons.org/licenses/by/4.0/ This content is distributed under the terms of the Creative Commons Attribution 4.0 International license.

10.1128/msystems.00169-22.3 FIG S3 Simulations of *Paenarthrobacter* TC1 metabolic performances in minimal media supplemented with different potential biostimulants. Performances are (A) growth, (B) atrazine degradation (mmol/g [dry weight]), and (C) consumption of biostimulant (mmol/g [dry weight]). Top and bottom panels represent supplementation with a standard amount of stimulant (50 mmol/g [dry weight]; top) or with fluxes normalized according to molecular nitrogen content (bottom). The initial amount of the biostimulants histidine and serine were 80 and 250 mmol/g [dry weight], respectively, the equal amount nitrogen to the atrazine. Download FIG S3, DOCX file, 0.09 MB. Copyright © 2022 Dhakar et al. 2022 Dhakar et al. https://creativecommons.org/licenses/by/4.0/ This content is distributed under the terms of the Creative Commons Attribution 4.0 International license.

10.1128/msystems.00169-22.8 TABLE S1 Effect of the total 109 metabolites on atrazine degradation. Download Table S1, DOCX file, 0.03 MB. Copyright © 2022 Dhakar et al. 2022 Dhakar et al. https://creativecommons.org/licenses/by/4.0/ This content is distributed under the terms of the Creative Commons Attribution 4.0 International license.

**FIG 2:**
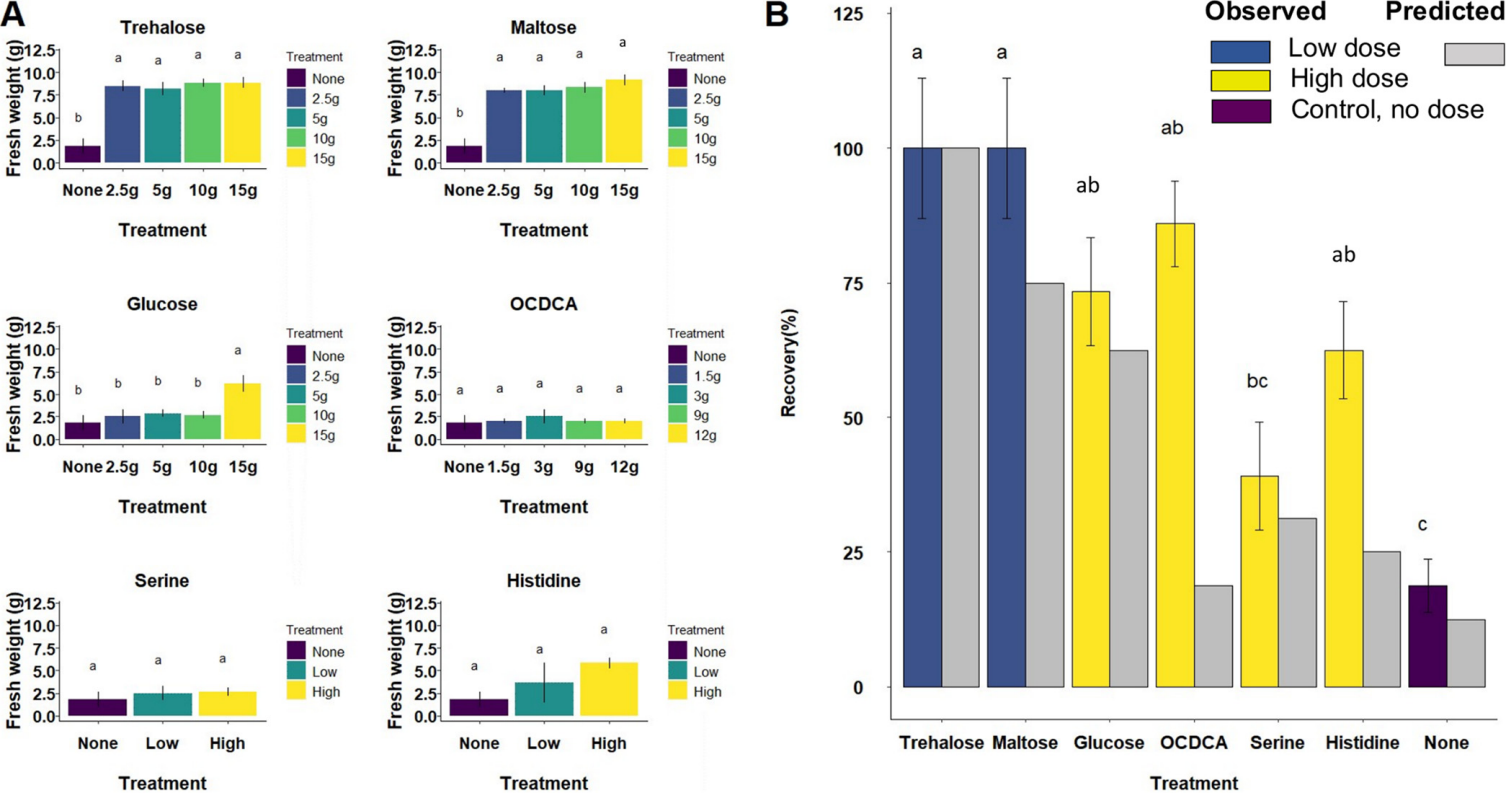
Pot experiments testing the effects of different supplements on atrazine persistence in soil. The level of atrazine was estimated according to the fresh weight of an atrazine-sensitive reporter plant. (A) Calibration experiment. Higher and lower doses of amino acids were as follows: histidine, 50 and 5 mg; serine, 104 and 10.4 mg per kg of soil. (B) Comparison between the predicted (gray bars) and observed (colored bars) biomass values in a follow-up experiment where each compound was provided in the minimal concentration that induced maximal effect (based on the calibration experiment). Coloring of the observed values is indicative of the selected dose of the metabolites, as indicated for the calibration experiment. Observed values are the recovery (percentage) of plant biomass, which was inversely proportional to the atrazine amount in the soil. Based on calibration experiments, trehalose and maltose were applied at a low dose (2.5 g per kg of soil), whereas other metabolites were applied at their respective high dose. Error bars represent standard errors.

Atrazine degradation in soil was evaluated in pot experiments, using an atrazine-sensitive weed for the bioassay (32). The potential enhancement effect of each of the stimulants was tested under four and two concentrations for the carbon sources and amino acids, respectively (Fig. 2A). A dose-sensitive effect was detected only for glucose, with trehalose and maltose reaching similar efficiencies with 2.5 g or 15 g per kg of soil. Based on the calibration results, biostimulants were compared considering the optimal minimal dose. Ranking of stimulant efficiency showed an overall agreement between model-based predictions and observations in pot experiments (Fig. 2B). Trehalose and maltose provided at a low dose (2.5 g per kg of soil) resulted in significantly higher recovery (*P* < 0.05) in comparison to glucose (at 15 g per kg of soil) and the other compounds tested. An exception was OCDCA, which had a similar biodegradation enhancement efficiency as glucose despite being predicted by the simulation to act as a poor enhancer.

### Impact of biostimulants on bacterial community structure.

The effect of biostimulants on the bacterial community structure was evaluated based on 16S rRNA gene amplicon sequences obtained from treated (with atrazine, supplements, and their respective combinations) and nontreated soil. Atrazine by itself did not induce a significant change in the diversity of soil bacteria, though it induced a significant difference in community structure (see Fig. S4), in correspondence with a previous report (32). Notably, each of the supplements and all combinations of atrazine and supplements induced a significant change in community structure with the exception of histidine (see Table S2). At the phylum level, *Proteobacteria* was the most abundant phylum (40 to 80%) across all treatments, with the highest relative abundance (>80%) in samples without any supplement (with or without atrazine) (see Fig. S5). Whereas no significant change was found at the phylum level between the control and atrazine, many of the supplements induced a clear shift in microbial composition that could be viewed across the phylogenetic tree (see Fig. S6). *Firmicutes* were supported by maltose, glucose, OCDCA, and histidine; Deltaproteobacteria were supported by maltose and glucose; and *Actinobacteria* were supported by trehalose and histidine. Most supplements suppressed *Gammaproteobacteria* species, with the exception of specific Pseudomonas species.

10.1128/msystems.00169-22.4 FIG S4 Effect of atrazine on microbial community structure. (A) Community diversity as indicated by alpha of Shannon’s index, comparing diversity between atrazine-treated and nontreated samples. Significance in diversity between atrazine treated and nontreated samples was determined with a Kruskal-Wallis rank-sum test requiring a *P* value of <0.05. (B) PCoA plots based on Bray-Curtis dissimilarity distances. (C) PERMANOVA testing the effect of atrazine and additives on the microbial community (1,000 permutations). The treatment groups are statistically significant at *P* < 0.05 on the basis of a nonparametric test. Download FIG S4, DOCX file, 0.6 MB. Copyright © 2022 Dhakar et al. 2022 Dhakar et al. https://creativecommons.org/licenses/by/4.0/ This content is distributed under the terms of the Creative Commons Attribution 4.0 International license.

10.1128/msystems.00169-22.5 FIG S5 Bacterial community structure at the phylum level. Download FIG S5, DOCX file, 0.1 MB. Copyright © 2022 Dhakar et al. 2022 Dhakar et al. https://creativecommons.org/licenses/by/4.0/ This content is distributed under the terms of the Creative Commons Attribution 4.0 International license.

10.1128/msystems.00169-22.6 FIG S6 (Left) Phylogenetic representation of the 16S bacterial community associated to naïve soil samples. (Right) Genera from which selected atrazine degraders were chosen are indicated by red circles; relative abundance is shown on the right graph. The location of *Geobacter* on the phylogenetic tree is highlighted through its upper taxonomic level (Deltaproteobacteria class) due to its low abundance. Download FIG S6, DOCX file, 1.1 MB. Copyright © 2022 Dhakar et al. 2022 Dhakar et al. https://creativecommons.org/licenses/by/4.0/ This content is distributed under the terms of the Creative Commons Attribution 4.0 International license.

10.1128/msystems.00169-22.9 TABLE S2 Beta diversity (pairwise Adonis test). Download Table S2, DOCX file, 0.02 MB. Copyright © 2022 Dhakar et al. 2022 Dhakar et al. https://creativecommons.org/licenses/by/4.0/ This content is distributed under the terms of the Creative Commons Attribution 4.0 International license.

Specific bacterial genera that were significantly affected by the biostimulants are listed in Table 1. As expected by simulations, growth of *Arthrobacter* was most strongly supported by trehalose and maltose, showing a significant increase in relative abundance. None of the other supplements that were predicted to have a weaker supportive effect on *P. aurescens* TC1 supported a significant increase in the relative abundance of *Arthrobacter*. Hence, the expediting of atrazine degradation by the optimal additives trehalose and maltose can be related to the significant increase in the relative abundance of *Arthrobacter*, which is not induced by atrazine *per se* (a weak enhancer of *Arthrobacter* growth) and is associated with a slower rate of degradation (Fig. 2).

**TABLE 1:** Differentially abundant bacterial genera in atrazine-treated samples versus samples treated with atrazine and supplement^*a*^

Genera differentially abundant after supplementation with:					
Maltose	Trehalose	Glucose	OCDCA	Serine	Histidine
*Adhaeribacter*	*Afifella*	*Anaeromyxobacter*	*Tepidibacter*	NA	*Pontibacter*
*Agromyces*	*Agromyces*	Arenimonas*			**Pseudomonas**
*Anaeromycbacter*	***Arthrobacter***	*Caloramator*			*Tepidibacter*
*Anaerovorax*	*Bradyrhizobium*	Cellvibrio*			
Arenimonas*	*Crocinitomix*	***Clostridium***			
***Arthrobacter***	*Flavisolibacter*	*Coprococcus*			
*Azoarcus*	*Geodermatophilus*	*Desulfosporosinus*			
*Bacillus*	*Labrys*	***Geobacter***			
*Balneimonas*	*Nitrospira*	*Parasegitibacter*			
*Bradyrhizobium*	*Phycicoccus*	*Steroidobacter*			
Cellvibrio*	*Rhodoplanes*	*Symbiobacterium*			
***Clostridium***	*Rubellimicrobium*				
*Dechloromonas*	*Sinorhizobium*				
***Geobacter***					
*Geodermatophilus*					
*Geosporobacter*					
Kaistobacter*					
*Kribella*					
*Labrys*					
Lysobacter*					
*Massilia*					
*Nitrospira*					
*Oxobacter*					
*Parasegitibacter*					
*Pontibacter*					
*Rubrobacter*					

a Genera whose relative abundance was reduced in the samples with supplement are indicated with an asterisk; other genera shown were significantly higher in abundance in the supplemented samples. Potential atrazine degraders according to a literature survey are shown in boldface. Differential abundance was determined using STAMP software and required a *P* value of <0.05 in a Welch test (following Benjamini-Hochberg FDR correction for multiple testing).

### Construction of a simulation matrix considering combinations of potential degraders and their stimulants.

**FIG 3:**
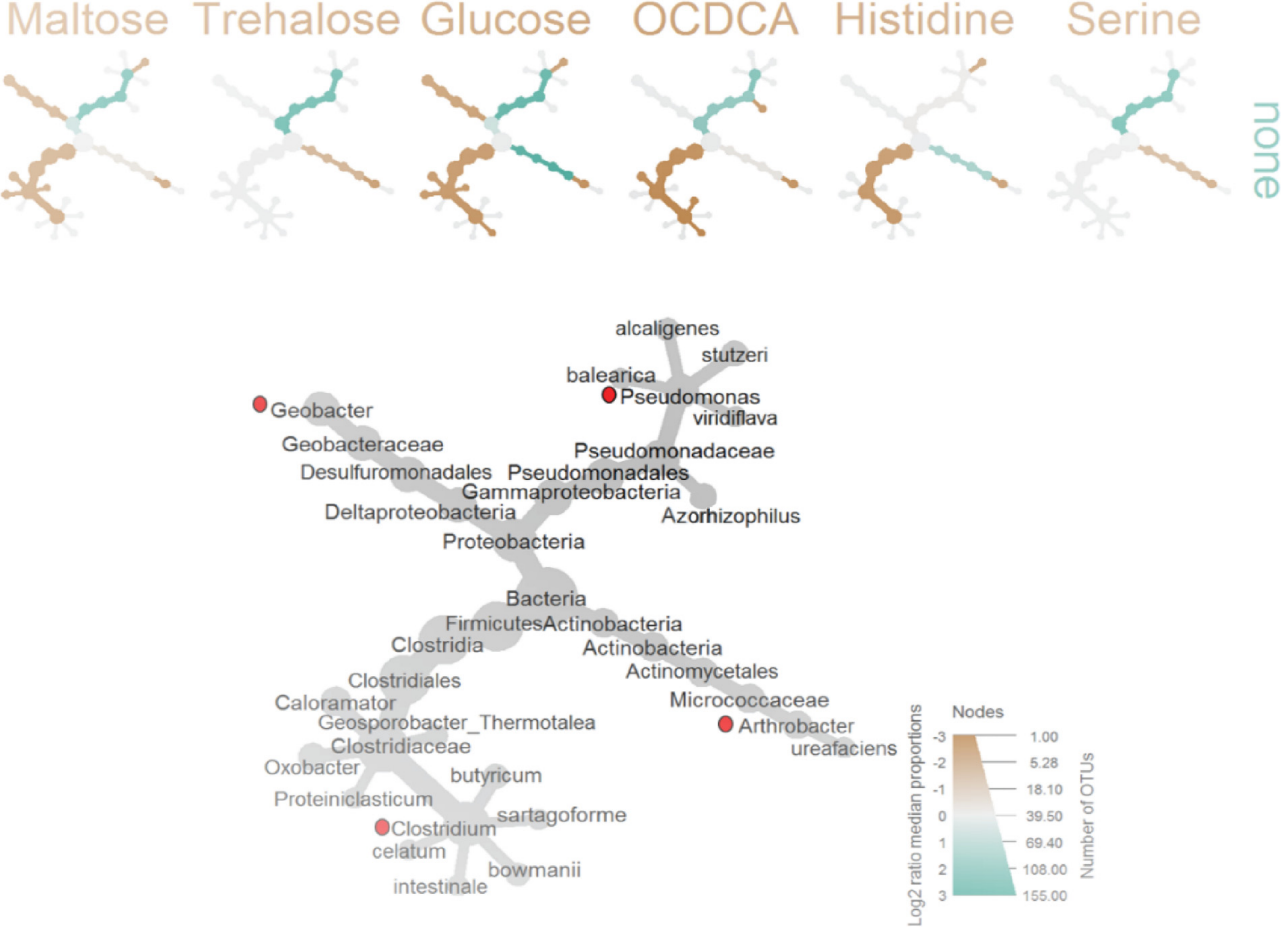
Effects of biostimulants on community structure. (Top) Differentially abundant bacterial groups in atrazine-treated samples supplemented with a biostimulant additive versus atrazine-only samples (“none”). Colors of the tree indicate higher abundance in the corresponding group, with significantly more microbial groups in the supplemented (blue) or not supplemented (brown) samples, as determined by a Wilcoxon test (*P* < 0.05) through its implementation in metacoder; these data were used for constructing the heat trees. (Bottom) List of differentially abundant bacterial genera. Differentially increased genera are indicated with an asterisk; other genera in the table were differentially decreased. Potential atrazine degraders according to a literature survey are shown in bold font. Differential abundance was determined using STAMP software.

Though the *Arthrobacter*-associated supplements maltose and trehalose were confirmed as the most efficient enhancers of degradation, other supplements (not associated with *Arthrobacter*) induced improved degradation in comparison to no-additive, control samples (Fig. 2B). Screening of the differentially abundant species induced in Newe-Ya’ar soil by these supplements pointed to several branches across the phylogenetic tree (Fig. 3). Assuming that the indigenous community contains several potential degraders of atrazine (either full or partial) that can be supported by different supplements, we aimed at constructing a two-dimensional simulation array, screening for the effect of potential biostimulants on a collection of potential indigenous degraders. Abundance data (Table 1) crossed with literature surveys led to the selection of three potential degraders representing taxonomic diversity: the *Proteobacteria* genera Pseudomonas and *Geobacter* (*Gammaproteobacteria* and Deltaproteobacteria, respectively) and the *Firmicutes* genus *Clostridium* (Fig. 3). Pseudomonas is a group well studied for its atrazine degradation activity, with a well-characterized six-gene pathway (42). *Geobacter* is widely known for its hydrocarbon degradation ability (43) and has also been suggested to participate in atrazine degradation (44). *Clostridium* is known as a potential degrader of atrazine (45) and has been reported to harbor genes associated with the atrazine degradation pathway (46). The abundance of these four bacterial genera in the native soil 16S bacterial community was as follows: *Arthrobacter* (7%) > Pseudomonas (0.5%) > *Clostridium* (0.2%) > *Geobacter* (0.02%) (see Fig. S6).

**FIG 4:**
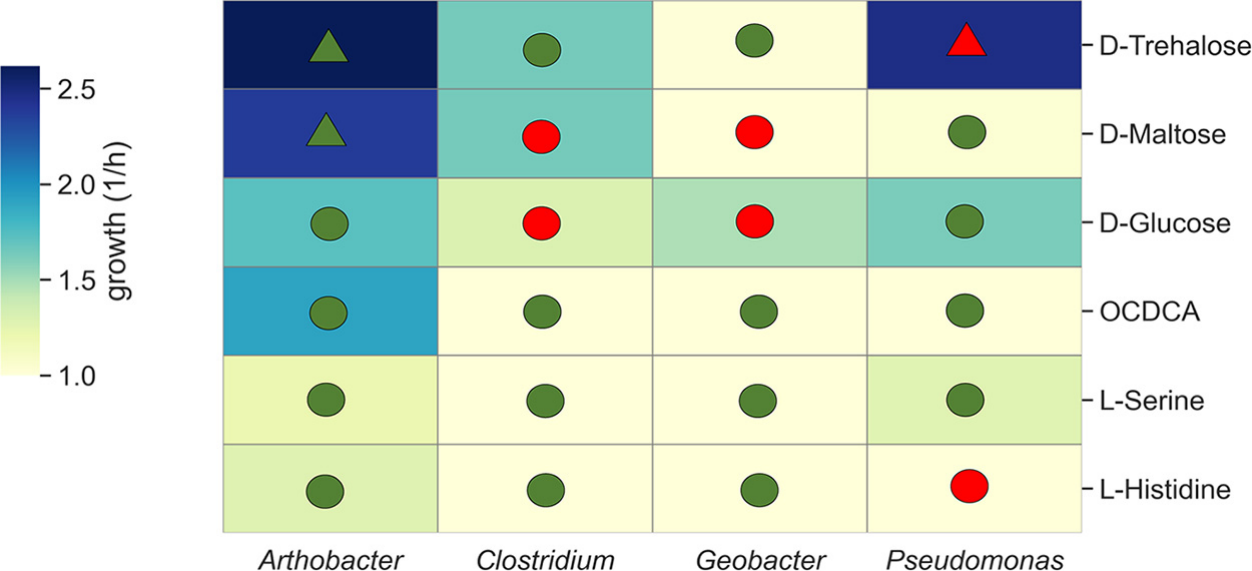
Construction of a simulation matrix testing the effect of biostimulants on potential native degraders. The matrix is the presentation of the relative growth of the species under the influence of biostimulants in a consortium of the four potential degraders (round 3). Values were normalized according to the fold change in each species in comparison to growth with no supplement. Triangles and circles represent positive and negative predictions, respectively, defined by at least a 2-fold increase in growth in comparison to the no-supplement group; green and red represent true and false observations, respectively, inferred from the significance of the increase in relative abundance (Table 1). Accuracy was calculated based on true positives (TP), true negatives (TN), false positives (FP), and false negatives (FN) as follows: (TP + TN)/(TP + TN + FP + FN) = 0.75. Precision was calculated as follows: TP/(FP + TP). Values for simulation outcomes and abundance in pot experiments are provided in Table S3 in the supplemental material.

Genomes for model construction were selected based on taxonomic proximities of sequenced species with the most abundant operational taxonomic units (OTUs) in the respective genera (see Fig. S2). Each of the selected genomes was found to include at least a single gene in pathways involved in atrazine degradation (Table 2). The selected Pseudomonas species exhibited 96% identity in the 16S rRNA gene to the Pseudomonas species P. stutzeri and P. putida (see Fig. S2), both reported as atrazine degraders (47, 48). Although atrazine degradation by *Geobacter* has not been reported, the genome of *Geobacter* (NC_011146) is known to harbor an amidohydrolase homologous with 55% identity to the ATZ/TRZ family chlorohydrolase (EHP89240), indicating a possible contribution to degradation. Model construction followed the procedure outlined in Xu et al. (32). Simulations compared the effect of each of the stimulants on an *in silico* consortium composed of the four potential degraders. Considering the community as a whole, the pattern of the simulations pointed to *Arthrobacter* as the main degrader, consistent with simulations of *Arthrobacter* alone, and pointed to trehalose and maltose as the optimal biostimulants (see Fig. S7), as inferred by the experimental observations. The simulation matrix in Fig. 4 points to the different relative effects of the biostimulants on the growth of each of the species within the consortium. Consistent with the 16S rRNA amplicon sequencing in the pot experiments, *Arthrobacter* is best supported by trehalose and maltose. The observations did not support the predictions for the support in trehalose in Pseudomonas (Fig. 4, false positive). The calculated precision and accuracy of the simulation matrix were 0.66 and 0.75, indicating an overall correspondence between predictions and observations regarding the influence of different biostimulants on different genera. The predicted and observed growth of the selected genera are shown in Table S3.

**TABLE 2:** Homologous proteins and genes present in genomes selected for model construction^*a*^

Genes encoding enzymes that take part in atrazine degradation	NCBI accession no.	Organism source of query proteins	Pseudomonas (NZ_CP026674.1)		*Clostridium* (NC_016791.1)		*Geobacter* (NC_011146.1)	
			% identity	QC	% identity	QC	% identity	QC
Atrazine ring								
*trzN*	ABM10554.1	Paenarthrobacter aurescens TC1	**33**	97	25	97	23	
*atzB*	ABM10408.1		**37**	89	25	92	32	
*atzC*	AAS20048.1		28	96	40	14		
*atzA*	AAK50270.1	Pseudomonas sp. ADP	28	97	**30**	93	25	
*atzB*	P95442.1		**38**	90	25	94	32	
*atzC*	O52063.1		28	96	40	14		
Isopropylamine								
*ipuA*	CAC81333.1	Pseudomonas sp. KIE171	25	91	24	90	25	89
*ipuB*	CAC81334.1		52	64	50	65	47	75
*ipuC*	CAC81335.1		**33**	99	98	28	28	97
*ipuD*	CAC81336.1		24	97				
*ipuE*	CAC81337.1						32	85
*ipuF*	CAC81338.1		31	57	26	64	30	49
*ipuG*	CAC81339.1		**31**	98				
*ipuH*	CAC81340.1		**57**	96	**48**	94	**34**	94
*ipuI*	CAC81341.1		**28**	87	27	83	24	85

a The known reactions were previously reported in Ofaim et al. (34). QC is the percent query coverage. Values in bold and underlined shared >30% homology and had >80% query coverage.

10.1128/msystems.00169-22.2 FIG S2 Phylogenetic relationships of the most abundant OTUs of the corresponding genera and the top hit in BLAST. The tree was constructed in MEGA 7.0 based on the neighbor-joining method with a bootstrap value of 1,000. Sequences in red and bold are for the selected genomes. Download FIG S2, DOCX file, 0.1 MB. Copyright © 2022 Dhakar et al. 2022 Dhakar et al. https://creativecommons.org/licenses/by/4.0/ This content is distributed under the terms of the Creative Commons Attribution 4.0 International license.

10.1128/msystems.00169-22.7 FIG S7 Simulations for biomass generation (A) and atrazine degradation (B) by community (all four member species) under the influence of biostimulants. Download FIG S7, DOCX file, 0.07 MB. Copyright © 2022 Dhakar et al. 2022 Dhakar et al. https://creativecommons.org/licenses/by/4.0/ This content is distributed under the terms of the Creative Commons Attribution 4.0 International license.

10.1128/msystems.00169-22.10 TABLE S3 (A) Predicted biomass of the individual microbial species in the model community supplemented with atrazine and biostimulants (one at a time). (B) Abundance of individual microbial groups retrieved from the 16S rRNA amplicon gene sequencing from the atrazine-treated groups supplemented with biostimulants. Download Table S3, DOCX file, 0.02 MB. Copyright © 2022 Dhakar et al. 2022 Dhakar et al. https://creativecommons.org/licenses/by/4.0/ This content is distributed under the terms of the Creative Commons Attribution 4.0 International license.

## DISCUSSION

As with many of the nutrimental fluxes in soil, biodegradation processes are often regulated by microbial communities. The activities of those complex assemblages can be manipulated, considering both their functioning (e.g., metabolic potential) and structure (relative abundance of critical taxa). Several studies have investigated the degradation performances of various degraders *in vitro*. However, it is difficult to get a full view of the degradation process in soil due to the interference of multiple additional biological components. “Omics” technologies have expanded the toolbox used for exploring the taxonomic and functional shifts in microbial communities and have been applied to explore the impact of aromatic pollutants and biostimulants (49). In particular, modeling approaches have been used to predict microbial community responses to environmental stimuli and for the optimization of targeted processes *in situ* (50). Such methods are cost-effective and reduce the time and efforts required for exploring complex systems such as soil, as well as bypass the inability of isolating the large majority of the native species (51).

Here, the model community was composed of four species predicted to be directly involved in degradation, each at a different rate. The application of biostimulants is known to alter the structure of a microbial community and subsequently lead to a change in the degradation rate of the targeted compound (52). The present study aimed at designing a biostimulation strategy that could enhance the biodegradation of the herbicide atrazine by targeting direct and indirect degrader taxa. Simulations were carried on two dimensions: screening for potential biostimulants versus the response of selected model species. Selected biostimulants were then validated in pot experiments. Among the screened biostimulants, trehalose and maltose were predicted and validated as the most significant enhancers of degradation, selectively targeting *Arthrobacter* species, the main degrader of atrazine in naive soil (i.e., with no prior exposure to atrazine). Nitrogenous biostimulants induced a weaker recovery in comparison to organic carbon-based biostimulants, in agreement with previous observations carried out with naive soils (53) . The overall abilities of the models to predict the compounds that act as taxa-selective stimulants indicate that computational algorithms can guide the manipulation of the soil microbiome.

In the current study, *Arthrobacter* was the only degrader supported by trehalose and maltose. Maltose supported several degraders, *Arthrobacter*, *Geobacter*, and *Clostridium*, with *Arthrobacter* the key degrader. Other compounds did not induce a significant increase in the relative abundance of *Arthrobacter*. In correspondence with previous studies, the addition of glucose reduced the growth of *Arthrobacter* in soil (21), although it might support alternative degraders. Here, *Clostridium* and *Geobacter* were among these other potential degraders, as also suggested in simulations. It has been reported that under some conditions, the biostimulants influence the resultant number of electrons (redox conditions) in the system associated with higher degradation rates (54, 55). Here, the contribution of glucose for the enhancement of atrazine degradation (though weaker than the direct biostimulants for *Arthrobacter*) can be associated with the growth of *Geobacter*, based on domination of the concept of bioelectrochemistry (44). An interlinked growth of *Clostridium* and *Geobacter* (also recognized as metal-reducing bacteria) was also reported in the biochar-amended soils described by Qiao et al. (56).

A key limitation of the current study is the construction of the metabolic models based on genomes that were retrieved from public repositories based on the identification of their taxonomy, as inferred from 16S amplicon sequencing of the respective samples. Such an approach might impose biases toward a limited collection of cultivated species that are not present in the native sample. Genome recovery, or genome-resolved metagenomics and often referred to as metagenome-assembled genomes, is an alternative approach that allows the construction of native genomes directly out of a metagenome. Such an approach is fundamentally superior over 16S-based genome computation, because the genomes are derived directly from the sample, without referring to a database, and allow an authentic look at the metabolic activities in native communities (57). Further improvement of model predictions might be gained from going beyond genomic and metagenomic data and considering additional multiomics data, i.e., metatranscriptomics, metaproteomics, and/or metabolomics (31). The integration of these additional layers of information into the model will further constrain the solution space and direct simulations toward feasible solutions. Mainly, such data will provide the currently missing information on the actual gene expression and protein activity that is not covered by the genomic information *per se*. Whereas the correspondence between predictions and observations is likely to be improved by the availability of data derived from the parallel profiling of samples with several multiomics technologies, other critical issues are currently unaddressed by most metabolic modeling approaches, including these used here, imposing challenges for future research. Key factors not encountered by the models included soil conditions that are not directly translated into nutritional content, such as pH and temperature, and nonmetabolic interactions such as quorum-sensing formation and toxin-mediated inhibition. Finally, scaling of the scope of simulations to communities and ecosystems poses numerous conceptual and technical uncertainties (31). Considering their scopes and limitations, the metabolic models can be viewed as tools for generating testable predictions through the contextualization of big data. Overall, the aim of the project was the development of a computational pipeline for the generation of model-based predictions for potential additives that will stimulate the degradation of atrazine by native soil communities and test these predictions in pot experiments, representing realistic conditions in a commercial field. Though currently limited to four genera, the construction of the simulation system provided a platform for expanding the simulation to additional species. These approaches can be easily adapted for the study of additional herbicides or other environmental pollutants as well. The work provides a pioneering application of metabolic modeling for the design of biostimulation strategies in soil and, more generally, in designing microbial community function in a complex environment. In particular, the pot experiments demonstrate that computational simulations can successfully rank the efficiency of different additives as potential biostimulants and relate between compounds and specific soil bacteria. Hence, this is a significant step forward in deciphering the black box of microbial function in a complex environment. Moreover, we have clearly demonstrated that processes in soil (i.e., herbicide degradation) are not optimized by themselves and are affected by the environmental conditions; hence, there is a promising potential for strategies that will allow harnessing the full potential of indigenous communities.

## MATERIALS AND METHODS

### Simulations to study the effects of supplements on atrazine degradation by P. aurescens TC1.

Simulations were based on an existing model of *P*. *aurescens* TC1 (34) and were carried using flux balance analysis (FBA), following protocols described by Dhakar et al. (33) and Ofaim et al. (36). As described by Dhakar et al. (33), the objective function was defined as growth through the maximization of the biomass reaction under different conditions. Flux variability analyses (FVA) (58) were carried to account for the possible flow of fluxes involved in secretion and uptake of metabolites. All simulations were carried out under defined conditions that follow experimentally verified viable conditions in minimal media with atrazine (34) and 109 exchange metabolites (one at a time) representing alternative carbon and nitrogen sources or other supplements (see Fig. S1 in the supplemental material). For each of the metabolite-supplemented media, dynamic modeling was used to predict the profile of biomass increase and atrazine degradation across time, as described previously (32–34). Briefly, the model works under the following assumptions: (i) a finite starting dose of medium components is available; (ii) a maximal amount of uptake that a single cell can acquire from the medium at a given time point is defined (the lower bound of the exchange reaction value); the maximal uptake was set to a ratio of ≤1 unit of each metabolite available in the medium per unit of biomass. (iii) After each time tick, the biomass amount was updated according to the flux amount of the biomass reaction in the model at this time tick. (iv) New substrate concentrations in each time point are determined by the predicted substrate concentration from the previous step augmented with any additional substrates secreted or consumed in the current iteration. The biomass production rate serves as a proxy for the size of the population in the simulated environment, and substrate uptake and secretion are mainly affected by the population size. Simulations were carried until reaching a state where additional time cycles did not lead to an increase in biomass.

All model simulations were done on an Intel i7 quad-core server with 128 GB of memory, running Linux. The development programming language of our simulators was Java, and our linear programming software was IBM CPLEX.

### Determining atrazine degradation in soil by using a bioassay plant reporter.

The simulated effect of selected biostimulants on microbial community was validated in pot experiments following the experimental procedure described by Xu et al. (32), using a bioassay atrazine-sensitive plant as a reporter for the rates of the herbicide in soil. Here, wheat (cv. Jordan) was used as the reporter plant, based on a demonstrated dose-dependent sensitivity of shoot development performance (biomass and height) to atrazine concentration in soil; growth performances are hence indicative of atrazine levels in soil. The bioassay experiments estimated atrazine degradation following soil amendment treatments in pots with soil from a non-herbicide-treated field. The soil amendments included combinations of atrazine and metabolites that are potential biostimulants. Experiments were carried out in replicates of five pots (0.5 liters), with 10 seeds sown in each. The soil was mixed with supplements by using a cement machine (Shatal; 150 liters), delivered into pots and sprayed with atrazine (1,000 g [active ingredient]/ha) on the soil surface. Atrazine herbicide (Atranex) was purchased from Adama Agan, Israel. Herbicides were applied using a motorized laboratory sprayer as described by Eizenberg et al. (59). The amounts of carbon-based biostimulants (maltose, trehalose, glucose, or OCDCA) applied at four concentrations were based on their carbon content (1, 2, 4, and 6 g of carbon/kg of soil), based on the methods of Xu et al. (32). Glucose monohydrate (≥99.5%) was purchased from Yishui Dadi Corn Development Co., Wujiawa, China. Maltose monohydrate (≥92%) was purchased from Thermo Fisher Scientific, UK. Trehalose dehydrate (≥98.0%) was purchased from TCI Tokyo Japan. Amino acids (≥98%) were purchased from Sigma-Aldrich. The amino acid biostimulants (histidine, serine glutamate, and leucine) were applied at two concentrations (14 and 1.4 mg of nitrogen/kg of soil), based on methods of Ofaim et al. (34). Leucine and glutamate were excluded from the study due to their inconsistent effects (data not shown).

Following calibration experiments, the experiment was repeated with the minimal concentration that supported maximal recovery, as determined for each compound in the calibration experiment. For each treatment, pots not treated with atrazine served as controls, in addition to no-atrazine–no-supplement pots and atrazine–no-supplement pots. Pots were irrigated as needed by sprinklers. The experiment was carried out in the net house during July to August (average maximum temperature 28°C to 32°C). The soils were collected 15 days after recording the effects of atrazine for all of the pots, and samples were frozen (−80°C) for further bacterial community analysis.

All experiments were arranged in a completely randomized design. One-way analysis of variance (ANOVA) computed the impact of herbicide phytotoxicity. Means were compared by a Tukey-Kramer honestly significant difference test (α, 0.05) using JMP software (version 7; SAS).

### DNA extraction, sequencing of 16S rRNA, and structure analysis of bacterial community.

Soil DNA was extracted by using a DNeasy Powerlyzer Powersoil kit (Qiagen) following the manufacturer’s instructions. The quality and quantity of the community DNA were checked through use of a Nanodrop apparatus (Thermo Scientific). The PCR system (Biometra) contained a total volume of 25 μL 2× PCR mixture and using *Taq* polymerase (Bio Ready mix; Bio-Lab), 5 μM primer (each), and 20 ng of DNA template. The PCR conditions were as follows: initial denaturation at 95°C for 3 min; 28 cycles of denaturation at 95°C for 30 s, primer annealing at 55°C for 30 s, and extension at 72°C for 45 s; followed by a final extension period of 10 min at 72°C. The amplicon sequencing was performed at the University of Illinois at Chicago Sequencing Core using MiSeq (Illumina). V3-V4 regions were amplified using the standard primer set 341F and 806R (32).

Quality control of the reads was carried out using the Quantitative Insights into Microbial Ecology platform (Qiime, version 2019.04) (60), with the plug-ins demux (https://github.com/qiime2/q2-demux) and dada2 (61). A total of 21,94,738 raw reads were reduced to 11,96,116 reads after the quality control, merging, and filtration steps (including denoising and chimera removal). The paired-end reads were combined based on overlapped regions, and the two 250-bp paired-end sequences were merged to obtain a single read (approximately 430 bp, mean length).

Taxonomic assignment of the resulting OTUs was done with q2_feature_classifier (62) using the Greengenes database (release 13_8) for 16S rRNA gene sequencing at 99% identity (63). Qiime2-generated files were converted to Phyloseq object element using the qiime2R package, and further analyses were done in Phyloseq (64). The feature table was pruned to remove the low-abundance OTUs (only sequences that appeared >5 times in at least half of the samples were included), and normal distribution was inferred using the Shapiro-Wilk test (*P* < 0.05). Differential abundance of OTUs was determined according to a Kruskal-Wallis rank sum test.

Summing up the OTUs into a higher-level taxonomy was conducted with STAMP (65) and its implemented Welch test (*P* < 0.05, following a Benjamini-Hochberg false-discovery rate adjustment) was used to determine differential abundance levels. Heat trees were constructed using metacoder (66).

### Reconstruction of metabolic network models for species representing selected genera and conducting community modeling.

Genome-scale metabolic models (GSMMs) were constructed for Pseudomonas, *Clostridium*, and *Geobacter* species. Genome sequences representing the respective genera in the native soil community were selected based on a BLAST search of the most highly abundant OTU in each of the selected genera versus public depositories of fully sequenced genomes. In cases where highly scored hits were retrieved for several species, the closest species for which a genome sequence was available were selected based on phylogenetic relatedness as inferred from a 16S rRNA-based phylogenetic tree (see Fig. S2). Representative OTUs and the respective genome sequences selected for constructing the metabolic networks models are listed in Table 3. The construction followed the protocol described by Xu et al. (32). Briefly, Model SEED was used for constructing the initial draft metabolic models from the genome sequence data (67). Annotations were done through RAST (68). After preparing a working draft model (that is, a biomass flux of >0 when all exchange reactions are open), each of the models was manually curated according to literature and other available resources, such as KEGG (69), UniProt (70), JGI (http://www.jgi.doe.gov/), and BiGG (71), to ensure that it captured the biochemical and physiological knowledge available. Steps in manual curation included updating of the draft model with new reactions retrieved from the additional annotation platforms, conversion of all reactions according to the KBASE (72) rxn conventions, validation of reaction stoichiometry and reversibility, and identification and elimination of futile loops. Finally, growth simulations were carried out in a minimal medium, in an iterative process ensuring that the reconstructed metabolic models were able to produce all biomass components in minimal mineral medium (MMM; K^+^, Mn^2+^, CO2, Zn^2+^, SO_4_^2−^, Cu^2+^, Ca^2+^, HPO_4_^2−^, Mg^2+^, Fe^2+^, Cl^−^) supplemented with alternative C and N sources, in accordance with species physiology. The final GSMM were consistent with experimental knowledge on the nutrients required for culturing each species (73–75).

**TABLE 3:** General features of the metabolic GSMM constructed for species representing highly abundant species in atrazine-treated soils

Feature	*P. aurescens* TC1	Pseudomonas sp. SWI44 chromosome	*Clostridium* sp. BNL1100	Geobacter bemidjiensis *bem*
Accession no.	NC_008711	NZ_CP026674	NC_016791	NC_011146
Taxonomy ID	290340	2083053	755731	404380
Identity with OTU^*a*^	98%	100%	93.1%	97.6%
Genome size (Mb)	5.23	5.92	4.61	4.62
No. of proteins	4,627	5,294	3,832	3,976
GSMM				
No. of proteins	960	977	592	669
Total no. of reactions	2,322	2,185	1,581	1,612
No. of biochemical reactions	2,047	1,580	1,076	1,185
No. of transport reactions	155	497	423	371
No. of exchange reactions	120	108	82	56
No. of metabolites	2,409	2,307	1,698	1,781

a Similarity between 16S rRNA of the OTU and the corresponding sequence in the genome.

Simulations were carried out using FBA following the same setting described above for the *P*. *aurescens* TC1 model. Community modeling was carried as described by Xu et al. (32) by joining together all species reconstructions. Briefly, our algorithm of community dynamic modeling uses dynamic FBA (dFBA) for simulating the growth of multiple species in a given medium across time (76, 77). The model is updated after each time tick. The amount of biomass of each species is changed after each time tick, based on the biomass reaction flux of the given species in that time tick; at each time point, we optimized the biomass flux for each species using the standard FBA optimization. Following each time tick, media uptake bounds and species biomass were updated to reflect secretions and uptakes of medium metabolites and biomass fluxes. The new concentrations were then used as a starting point for the next iteration. Simulations assumed equal initial biomass for each species to gain a qualitative view of the effect of each potential biostimulant. A detailed description of the algorithm is available in data file S6 via Figshare (https://figshare.com/s/a7b20190119c745c1fa1).

Models are available as Systems Biology Markup Language (SBML) files (78) in supplementary data files S1 to S4 available via Figshare. The SBML file can be used with tools such as MATLAB or other SBML-compliant software. Simulation definitions, running conditions, and simulation outcomes applied for predicting the effect of supplements on atrazine degradation by Paenarthrobacter aurescens TC1 (Fig. 1) are provided in supplementary data file S5 via Figshare. Simulation conditions, model definitions, running algorithm, and simulation outcomes applied for predicting the effect of supplements on atrazine degradation by Paenarthrobacter aurescens TC1, Pseudomonas, *Clostridium*, and *Geobacter* (Fig. 4) are provided in supplementary data file S6 via Figshare.
