# Supplementary material for: Modeling-Guided Amendments Lead to Enhanced Biodegradation in Soil
Source: mSystems. 2022 Aug 1;7(4):e00169-22. doi: 10.1128/msystems.00169-22 (PMC9426591; doi:10.1128/msystems.00169-22)
Supplement: FIG S1 [file msystems.00169-22-s0001.docx]

**Modelling-guided amendments lead to enhanced biodegradation in soil**

Kusum Dhakar^1,2¥^, Raphy Zarecki^1,2¥^, Shlomit Medina^1^, Hamam Ziadna^1^, Karam Igbaria^1^, Ran Lati^1^, Zeev Ronen^2 ϯ^, Hanan Eizenberg^1^ & Shiri Freilich^1^*^ϯ^

^1^Newe Ya'ar Research Center, Agricultural Research Organization, Ramat Yishay, Israel, ^2^Department of Environmental Hydrology & Microbiology, Zuckerberg Institute for Water Research, Jacob Blaustein Institutes for Desert Research, Ben-Gurion University of the Negev, Midreshet Ben-Gurion, 8499000, Israel,

^3^Albert Katz School for Desert Studies Jacob Blaustein Institutes for Desert Research, Ben-Gurion University of the Negev, Midreshet Ben-Gurion, 8499000, Israel,

^4^Junior Research Group Microbial Biotechnology, Leibniz Institute DSMZ, German Collection of Microorganisms and Cell Cultures, Braunschweig, Germany

^¥^equal contribution

^ϯ^ equal contribution

* Corresponding author (shiri@agri.gov.il,+972506220047)


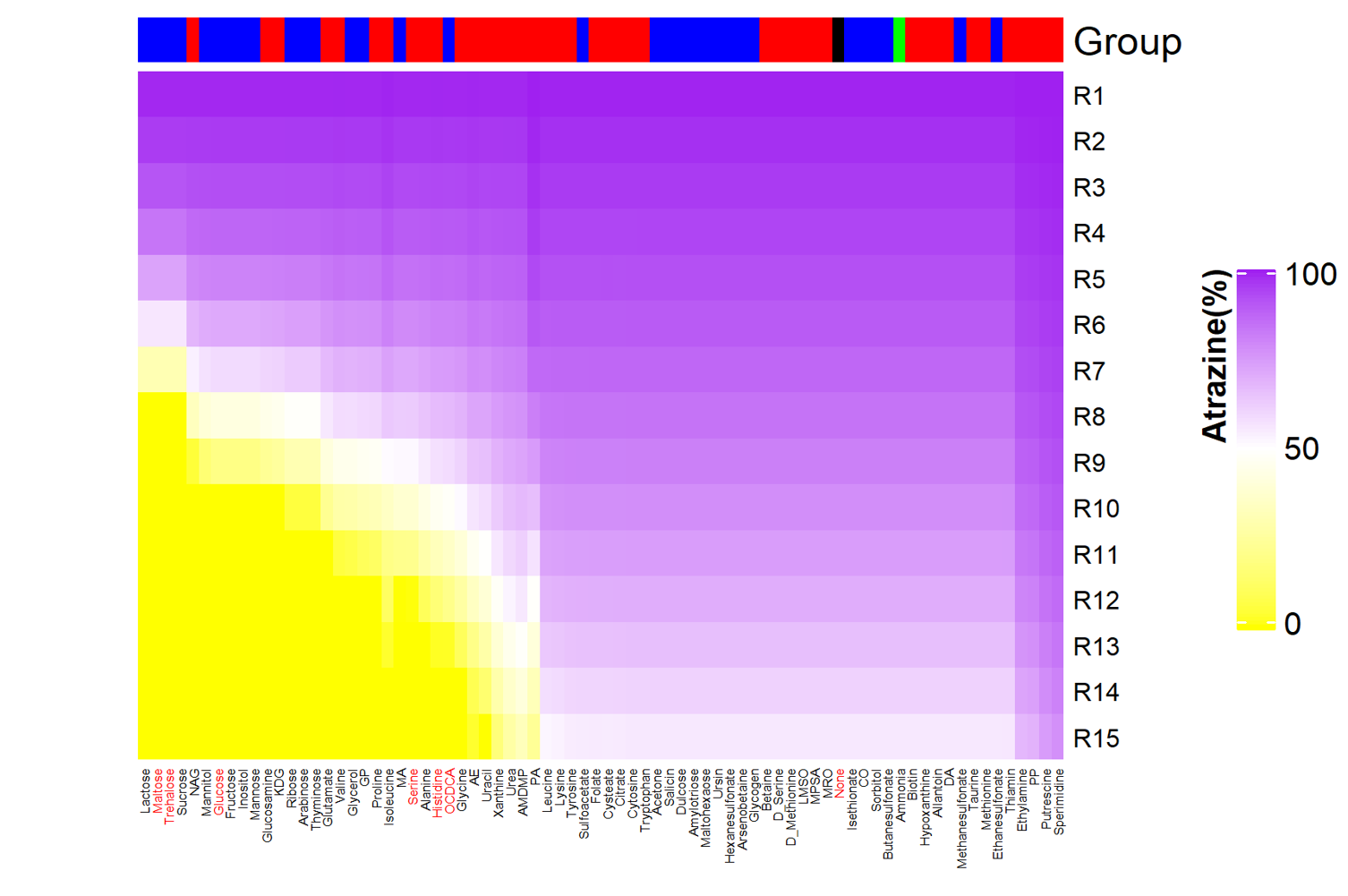


**Fig.S1.**

We simulated growth in 109 different media combinations, each supplementing the atrazine containing minimal mineral media with a single exchange metabolite. Out of 109 potential supplements, total 75 metabolites were retained after eliminating non practical additives such as dipeptides and toxic substances from the initial set of exchange metabolites. Group represents: Blue= Carbon source; Red = Both carbon and nitrogen source; Green = Nitrogen source and Black = None as no supplement. Rows are representing rounds (1-15). Atrazine amount is represented from 100 to 0 as purple to yellow, respectively. NAG= N-Acetyl-D-Glucosamine; KDG=2-Keto-3-deoxy-D-gluconate; GP=Glycerol-3-Phosphate; MA=Myristic acid; AE= Aminoethanol; PA = L-Phenyl alanine; DA= 5'-Deoxyadenosine; LMSO= L-Methionine-S-oxide; MPSA=morpholinopropane sulfonic acid; MRO=L-methionine R-oxide; CO=Carbon mono oxide; PP=2-Propanamine.
