# Supplementary material for: Modeling-Guided Amendments Lead to Enhanced Biodegradation in Soil
Source: mSystems. 2022 Aug 1;7(4):e00169-22. doi: 10.1128/msystems.00169-22 (PMC9426591; doi:10.1128/msystems.00169-22)
Supplement: TABLE S1 [file msystems.00169-22-s0008.docx]

**Modelling-guided amendments lead to enhanced biodegradation in soil**

Kusum Dhakar^1,2¥^, Raphy Zarecki^1,2¥^, Shlomit Medina^1^, Hamam Ziadna^1^, Karam Igbaria^1^, Ran Lati^1^, Zeev Ronen^2 ϯ^, Hanan Eizenberg^1^ & Shiri Freilich^1^*^ϯ^

^1^Newe Ya'ar Research Center, Agricultural Research Organization, Ramat Yishay, Israel, ^2^Department of Environmental Hydrology & Microbiology, Zuckerberg Institute for Water Research, Jacob Blaustein Institutes for Desert Research, Ben-Gurion University of the Negev, Midreshet Ben-Gurion, 8499000, Israel,

^3^Albert Katz School for Desert Studies Jacob Blaustein Institutes for Desert Research, Ben-Gurion University of the Negev, Midreshet Ben-Gurion, 8499000, Israel,

^4^Junior Research Group Microbial Biotechnology, Leibniz Institute DSMZ, German Collection of Microorganisms and Cell Cultures, Braunschweig, Germany

^¥^equal contribution

^ϯ^ equal contribution

* Corresponding author (shiri@agri.gov.il,+972506220047)

**Table S1. Effect of total 109 metabolites on atrazine degradation**

| Round | Total_Biomass | Atrazine_left | CPD | Compound Name |
| --- | --- | --- | --- | --- |
| 8 | 27.41741972 | 1.30E-13 | cpd00208 | Lactose |
| 8 | 27.41741972 | 4.12E-13 | cpd00179 | Maltose |
| 8 | 27.41741972 | 2.32E-12 | cpd00794 | D-Trehalose |
| 8 | 27.41741972 | 1.02E-11 | cpd00076 | Sucrose |
| 8 | 14.24172047 | 16.37309146 | cpd00122 | N-Acetyl-D-Glucosamine |
| 8 | 12.12658299 | 19.60383792 | cpd00314 | D-Mannitol |
| 8 | 11.36085799 | 20.81117432 | cpd00027 | D-Glucose |
| 8 | 11.36085799 | 20.81117433 | cpd00121 | L-Inositol |
| 8 | 11.360858 | 20.81117433 | cpd00082 | D-Fructose |
| 8 | 11.36085799 | 20.81117433 | cpd00138 | D-Mannose |
| 8 | 11.23843863 | 21.00627571 | cpd11586 | Ala-L-Glu-L |
| 8 | 10.6377789 | 21.97238488 | cpd00276 | D-Glucosamine |
| 8 | 10.33934728 | 22.45808435 | cpd11582 | Ala-L-Thr-L |
| 8 | 10.28768476 | 22.54256574 | cpd00176 | 2-Keto-3-deoxy-D-gluconate |
| 8 | 9.86216179 | 23.24305914 | cpd11587 | Ala-Gln |
| 8 | 9.311432508 | 24.16260263 | cpd01242 | Thyminose |
| 8 | 9.311432508 | 24.16260263 | cpd00224 | L-Arabinose |
| 8 | 9.311432508 | 24.16260263 | cpd00105 | D-Ribose |
| 8 | 8.993483716 | 24.7005519 | cpd11592 | Gly-Glu-L |
| 8 | 8.993483716 | 24.7005519 | cpd11593 | Ala-L-Asp-L |
| 8 | 8.544206002 | 25.47115802 | cpd11588 | Gly-Pro-L |
| 8 | 8.140557661 | 26.17161505 | cpd11580 | Gly-Gln |
| 8 | 7.753694472 | 26.85449153 | cpd11584 | Ala-His |
| 8 | 7.319326158 | 27.63101665 | cpd00023 | L-Glutamate |
| 8 | 7.151164165 | 27.93571123 | cpd11589 | Gly-Asp-L |
| 8 | 6.687887495 | 28.78621537 | cpd11581 | Gly-Asn-L |
| 8 | 6.608042354 | 28.93454117 | cpd00100 | Glycerol |
| 8 | 7.185037215 | 29.09714638 | cpd00156 | L-Valine |
| 8 | 6.362325181 | 29.39439922 | cpd00080 | Glycerol-3-Phosphate |
| 8 | 6.362325178 | 29.39439922 | cpd11585 | L-Alanylglycine |
| 8 | 6.328336472 | 29.67924418 | cpd00129 | L-Proline |
| 8 | 11.59686764 | 30.92155184 | cpd15605 | Gly-Phe |
| 8 | 5.483940535 | 31.08627937 | cpd03847 | Myristic acid |
| 8 | 5.467306061 | 31.11702024 | cpd00054 | Serine |
| 8 | 8.057794035 | 31.55275231 | cpd00322 | L-Isoleucine |
| 8 | 4.933723563 | 32.18576612 | cpd00117 | D-Alanine |
| 8 | 4.825318708 | 33.16235273 | cpd00119 | Histidine |
| 8 | 4.34342451 | 33.41324863 | cpd01080 | OCDCA |
| 8 | 4.001497553 | 34.14110854 | cpd00033 | Glycine |
| 8 | 3.991207991 | 35.82500332 | cpd00162 | Aminoethanol |
| 8 | 3.233972726 | 35.86396889 | cpd00092 | Uracil |
| 8 | 3.27599376 | 36.15755669 | cpd15606 | Gyl-Tyr |
| 8 | 3.031253865 | 36.33898007 | cpd00007 | Oxygen |
| 8 | 2.735846295 | 37.05182918 | cpd00209 | Nitrate |
| 8 | 2.735846302 | 37.05284356 | cpd00309 | Xanthine |
| 8 | 2.445868605 | 37.7778503 | cpd00073 | Urea |
| 8 | 2.445868598 | 37.77785039 | cpd00075 | Nitrite |
| 7 | 2.053945529 | 40.26962311 | cpd02894 | 4-Amino-2-methyl-5-diphosphomethylpyrimidine |
| 8 | 8.131890007 | 40.38466085 | cpd00066 | L-Phenyl alanine |
| 8 | 2.283443125 | 41.28607484 | cpd15604 | Gly-Leu |
| 8 | 2.299680554 | 41.34614091 | cpd11583 | Ala-Leu |
| 8 | 2.23428344 | 41.51588755 | cpd00107 | L-Leucine |
| 8 | 2.187614936 | 41.60163846 | cpd00039 | L-Lysine |
| 8 | 2.134188062 | 41.71441673 | cpd00069 | L-Tyrosine |
| 8 | 2.115229153 | 41.72658897 | cpd09878 | Sulfoacetate |
| 8 | 2.111387175 | 41.74944776 | cpd00393 | Folate |
| 8 | 2.116859473 | 41.75530929 | cpd00395 | L-Cysteate |
| 8 | 2.100933636 | 41.75542433 | cpd00137 | Ctirate |
| 8 | 2.194908494 | 41.79188293 | cpd00067 | Proton |
| 8 | 2.093699458 | 41.80990787 | cpd00307 | Cytosine |
| 8 | 2.090276267 | 41.82206916 | cpd00971 | Na+ |
| 8 | 2.090276267 | 41.82206916 | cpd03696 | Ursin |
| 8 | 2.090276267 | 41.82206916 | cpd00540 | Betaine |
| 8 | 2.090276267 | 41.82206917 | cpd15302 | Glycogen(n-1) |
| 8 | 2.090276267 | 41.82206917 | cpd01012 | Cd+2 |
| 8 | 2.090276267 | 41.82206917 | cpd00178 | Acetone |
| 8 | 2.090276267 | 41.82206917 | cpd00244 | Ni+2 |
| 8 | 2.090276267 | 41.82206917 | cpd00531 | Hg+2 |
| 8 | 2.090276267 | 41.82206917 | cpd00550 | D-Serine |
| 8 | 2.090276267 | 41.82206917 | cpd00637 | D-Methionine |
| 8 | 2.090276267 | 41.82206917 | cpd01030 | Salicin |
| 8 | 2.090276267 | 41.82206917 | cpd01171 | Dulcose |
| 8 | 2.090276267 | 41.82206917 | cpd01262 | Amylotriose |
| 8 | 2.090276267 | 41.82206917 | cpd01329 | Maltohexaose |
| 8 | 2.090276267 | 41.82206917 | cpd01914 | L-Methionine-S-oxide |
| 8 | 2.090276267 | 41.82206917 | cpd03724 | Ferrichrom |
| 8 | 2.090276267 | 41.82206917 | cpd04097 | Ph |
| 8 | 2.090276267 | 41.82206917 | cpd11575 | morpholinopropane sulfonic acid |
| 8 | 2.090276267 | 41.82206917 | cpd11576 | L-methionine R-oxide |
| 8 | 2.090276267 | 41.82206917 | cpd11578 | Hexanesulfonate |
| 8 | 2.090276267 | 41.82206917 | cpd11597 | Arsenobetaine |
| 8 | 2.090276267 | 41.82206917 | none | None |
| 8 | 2.090276264 | 41.8220692 | cpd00588 | Sorbitol |
| 8 | 2.090276264 | 41.8220692 | cpd00204 | Carbon mono oxide |
| 8 | 2.090276263 | 41.82206921 | cpd03048 | Isethionate |
| 8 | 2.090276263 | 41.82206921 | cpd00226 | Hypoxanthine |
| 8 | 2.090276263 | 41.82206921 | cpd03091 | 5'-Deoxyadenosine |
| 8 | 2.090276263 | 41.82206921 | cpd00013 | Ammonia |
| 8 | 2.090276263 | 41.82206921 | cpd00104 | Biotin |
| 8 | 2.090276263 | 41.82206921 | cpd01092 | Allantoin |
| 8 | 2.090276263 | 41.82206921 | cpd11416 | Biomass |
| 8 | 2.090276263 | 41.82206921 | cpd00012 | Diphosphate |
| 8 | 2.090276263 | 41.82206921 | cpd00011 | Carbon di oxide |
| 8 | 2.090276263 | 41.82206921 | cpd11596 | Butanesulfonate |
| 8 | 2.100014523 | 41.82343967 | cpd00028 | Heme |
| 8 | 2.090182396 | 41.82393008 | cpd08023 | Methanesulfonate |
| 8 | 2.090182395 | 41.82472426 | cpd00210 | Taurine |
| 8 | 2.105981663 | 41.82478987 | cpd00065 | L-Tryptophan |
| 8 | 2.090182395 | 41.8258292 | cpd00060 | L-Methionine |
| 8 | 2.090182395 | 41.82790096 | cpd11579 | Ethanesulfonate |
| 8 | 2.090182395 | 41.82790096 | cpd01017 | Cys-Gly |
| 8 | 2.090182395 | 41.82790096 | cpd15603 | Gly-Cys |
| 8 | 2.091887689 | 41.84107505 | cpd00305 | Thiamin |
| 8 | 2.096543795 | 42.16577021 | cpd11591 | Gly-Met |
| 8 | 2.100232188 | 42.19470274 | cpd11590 | Met-L-Ala-L |
| 8 | 2.724601673 | 44.92843462 | cpd00591 | Ethylamine |
| 8 | 2.755046093 | 45.14565836 | cpd04129 | 2-Propanamine |
| 8 | 2.755046152 | 45.96403756 | cpd00118 | Putrescine |
| 8 | 2.755046123 | 46.42168739 | cpd00264 | Spermidine |
