# Supplementary material for: Modeling-Guided Amendments Lead to Enhanced Biodegradation in Soil
Source: mSystems. 2022 Aug 1;7(4):e00169-22. doi: 10.1128/msystems.00169-22 (PMC9426591; doi:10.1128/msystems.00169-22)
Supplement: TABLE S2 [file msystems.00169-22-s0009.docx]

**Modelling-guided amendments lead to enhanced biodegradation in soil**

Kusum Dhakar^1,2¥^, Raphy Zarecki^1,2¥^, Shlomit Medina^1^, Hamam Ziadna^1^, Karam Igbaria^1^, Ran Lati^1^, Zeev Ronen^2 ϯ^, Hanan Eizenberg^1^ & Shiri Freilich^1^*^ϯ^

^1^Newe Ya'ar Research Center, Agricultural Research Organization, Ramat Yishay, Israel, ^2^Department of Environmental Hydrology & Microbiology, Zuckerberg Institute for Water Research, Jacob Blaustein Institutes for Desert Research, Ben-Gurion University of the Negev, Midreshet Ben-Gurion, 8499000, Israel,

^3^Albert Katz School for Desert Studies Jacob Blaustein Institutes for Desert Research, Ben-Gurion University of the Negev, Midreshet Ben-Gurion, 8499000, Israel,

^4^Junior Research Group Microbial Biotechnology, Leibniz Institute DSMZ, German Collection of Microorganisms and Cell Cultures, Braunschweig, Germany

^¥^equal contribution

^ϯ^ equal contribution

* Corresponding author (shiri@agri.gov.il,+972506220047)

**Table S2. Beta diversity (Pairwise Adonis Test)**

| S.No. | Pairs | R2 | p.value | p.adjusted |
| --- | --- | --- | --- | --- |
| 1 | Cn_none vs Cn_Trehalose | 0.770278 | 0.01 | 0.016901 |
| 2 | Cn_none vs Az_Trehalose | 0.790854 | 0.01 | 0.016901 |
| 3 | Cn_none vs Cn_Glucose | 0.713924 | 0.004 | 0.016901 |
| 4 | Cn_none vs Cn_Maltose | 0.851848 | 0.005 | 0.016901 |
| 5 | Cn_none vs Cn_OCDCA | 0.818134 | 0.009 | 0.016901 |
| 6 | Cn_none vs Cn_time0 | 0.8443 | 0.008 | 0.016901 |
| 7 | Cn_none vs Az_time0 | 0.568641 | 0.009 | 0.016901 |
| 8 | Az_none vs Cn_Trehalose | 0.659165 | 0.004 | 0.016901 |
| 9 | Az_none vs Az_Trehalose | 0.666599 | 0.01 | 0.016901 |
| 10 | Az_none vs Cn_Glucose | 0.593649 | 0.007 | 0.016901 |
| 11 | Az_none vs Cn_Maltose | 0.742494 | 0.004 | 0.016901 |
| 12 | Az_none vs Az_Maltose | 0.750691 | 0.006 | 0.016901 |
| 13 | Az_none vs Cn_OCDCA | 0.723035 | 0.006 | 0.016901 |
| 14 | Az_none vs Az_OCDCA | 0.586644 | 0.008 | 0.016901 |
| 15 | Az_none vs Az_time0 | 0.525517 | 0.008 | 0.016901 |
| 16 | Cn_Trehalose vs Cn_Glucose | 0.54739 | 0.01 | 0.016901 |
| 17 | Cn_Trehalose vs Az_Glucose | 0.616299 | 0.007 | 0.016901 |
| 18 | Cn_Trehalose vs Cn_Maltose | 0.338702 | 0.004 | 0.016901 |
| 19 | Cn_Trehalose vs Az_Maltose | 0.4247 | 0.008 | 0.016901 |
| 20 | Cn_Trehalose vs Cn_OCDCA | 0.316209 | 0.007 | 0.016901 |
| 21 | Cn_Trehalose vs Cn_Histidine | 0.375971 | 0.009 | 0.016901 |
| 22 | Cn_Trehalose vs Az_Serine | 0.555707 | 0.007 | 0.016901 |
| 23 | Cn_Trehalose vs Cn_time0 | 0.565808 | 0.004 | 0.016901 |
| 24 | Az_Trehalose vs Az_Glucose | 0.614969 | 0.009 | 0.016901 |
| 25 | Az_Trehalose vs Cn_Maltose | 0.530157 | 0.009 | 0.016901 |
| 26 | Az_Trehalose vs Az_Maltose | 0.586415 | 0.009 | 0.016901 |
| 27 | Az_Trehalose vs Cn_OCDCA | 0.478815 | 0.008 | 0.016901 |
| 28 | Az_Trehalose vs Cn_Histidine | 0.404872 | 0.01 | 0.016901 |
| 29 | Az_Trehalose vs Az_Histidine | 0.771831 | 0.008 | 0.016901 |
| 30 | Az_Trehalose vs Az_Serine | 0.580049 | 0.004 | 0.016901 |
| 31 | Az_Trehalose vs Az_time0 | 0.361339 | 0.006 | 0.016901 |
| 32 | Cn_Glucose vs Cn_Maltose | 0.625557 | 0.008 | 0.016901 |
| 33 | Cn_Glucose vs Az_Maltose | 0.594082 | 0.008 | 0.016901 |
| 34 | Cn_Glucose vs Cn_OCDCA | 0.585532 | 0.008 | 0.016901 |
| 35 | Cn_Glucose vs Cn_Histidine | 0.473178 | 0.004 | 0.016901 |
| 36 | Cn_Glucose vs Az_Histidine | 0.665546 | 0.009 | 0.016901 |
| 37 | Cn_Glucose vs Cn_Serine | 0.385667 | 0.008 | 0.016901 |
| 38 | Cn_Glucose vs Az_Serine | 0.536427 | 0.007 | 0.016901 |
| 39 | Cn_Glucose vs Az_time0 | 0.434924 | 0.008 | 0.016901 |
| 40 | Az_Glucose vs Cn_Maltose | 0.681152 | 0.004 | 0.016901 |
| 41 | Az_Glucose vs Az_Maltose | 0.671609 | 0.005 | 0.016901 |
| 42 | Az_Glucose vs Cn_OCDCA | 0.664133 | 0.005 | 0.016901 |
| 43 | Az_Glucose vs Az_time0 | 0.517459 | 0.006 | 0.016901 |
| 44 | Cn_Maltose vs Az_Maltose | 0.250561 | 0.006 | 0.016901 |
| 45 | Cn_Maltose vs Cn_OCDCA | 0.302301 | 0.009 | 0.016901 |
| 46 | Cn_Maltose vs Az_OCDCA | 0.397294 | 0.01 | 0.016901 |
| 47 | Cn_Maltose vs Cn_Histidine | 0.541924 | 0.007 | 0.016901 |
| 48 | Cn_Maltose vs Az_Histidine | 0.808694 | 0.009 | 0.016901 |
| 49 | Cn_Maltose vs Az_Serine | 0.703851 | 0.009 | 0.016901 |
| 50 | Cn_Maltose vs Cn_time0 | 0.542819 | 0.009 | 0.016901 |
| 51 | Cn_Maltose vs Az_time0 | 0.31575 | 0.009 | 0.016901 |
| 52 | Az_Maltose vs Az_OCDCA | 0.395667 | 0.009 | 0.016901 |
| 53 | Az_Maltose vs Cn_Histidine | 0.596502 | 0.009 | 0.016901 |
| 54 | Az_Maltose vs Az_Histidine | 0.792573 | 0.01 | 0.016901 |
| 55 | Az_Maltose vs Cn_Serine | 0.481576 | 0.002 | 0.016901 |
| 56 | Az_Maltose vs Az_Serine | 0.704953 | 0.009 | 0.016901 |
| 57 | Az_Maltose vs Cn_time0 | 0.468492 | 0.01 | 0.016901 |
| 58 | Cn_OCDCA vs Az_OCDCA | 0.355623 | 0.009 | 0.016901 |
| 59 | Cn_OCDCA vs Cn_Histidine | 0.522038 | 0.008 | 0.016901 |
| 60 | Cn_OCDCA vs Az_Histidine | 0.78058 | 0.01 | 0.016901 |
| 61 | Cn_OCDCA vs Az_Serine | 0.663788 | 0.009 | 0.016901 |
| 62 | Cn_OCDCA vs Cn_time0 | 0.515377 | 0.01 | 0.016901 |
| 63 | Cn_OCDCA vs Az_time0 | 0.289489 | 0.008 | 0.016901 |
| 64 | Az_OCDCA vs Az_Histidine | 0.577173 | 0.01 | 0.016901 |
| 65 | Az_OCDCA vs Cn_time0 | 0.480138 | 0.01 | 0.016901 |
| 66 | Cn_Histidine vs Az_Histidine | 0.449275 | 0.008 | 0.016901 |
| 67 | Cn_Histidine vs Cn_time0 | 0.632671 | 0.009 | 0.016901 |
| 68 | Az_Histidine vs Cn_time0 | 0.811016 | 0.006 | 0.016901 |
| 69 | Cn_Serine vs Cn_time0 | 0.524325 | 0.008 | 0.016901 |
| 70 | Az_Serine vs Az_time0 | 0.403775 | 0.007 | 0.016901 |
| 71 | Cn_time0 vs Az_time0 | 0.346681 | 0.008 | 0.016901 |
| 72 | Az_none vs Az_Glucose | 0.394388 | 0.011 | 0.016923 |
| 73 | Az_none vs Cn_time0 | 0.758335 | 0.011 | 0.016923 |
| 74 | Cn_Trehalose vs Az_Histidine | 0.739775 | 0.011 | 0.016923 |
| 75 | Az_Trehalose vs Cn_time0 | 0.68333 | 0.011 | 0.016923 |
| 76 | Az_Maltose vs Cn_OCDCA | 0.339809 | 0.011 | 0.016923 |
| 77 | Az_Histidine vs Az_time0 | 0.557102 | 0.011 | 0.016923 |
| 78 | Az_Serine vs Cn_time0 | 0.722631 | 0.011 | 0.016923 |
| 79 | Cn_none vs Az_Maltose | 0.841455 | 0.012 | 0.017349 |
| 80 | Cn_Trehalose vs Az_Trehalose | 0.294647 | 0.012 | 0.017349 |
| 81 | Cn_Glucose vs Az_Glucose | 0.561695 | 0.012 | 0.017349 |
| 82 | Cn_Glucose vs Az_OCDCA | 0.47291 | 0.012 | 0.017349 |
| 83 | Cn_Maltose vs Cn_Serine | 0.410571 | 0.012 | 0.017349 |
| 84 | Cn_Trehalose vs Az_OCDCA | 0.342366 | 0.013 | 0.018353 |
| 85 | Az_Trehalose vs Cn_Glucose | 0.513945 | 0.013 | 0.018353 |
| 86 | Az_Trehalose vs Az_OCDCA | 0.422133 | 0.014 | 0.018876 |
| 87 | Cn_Glucose vs Cn_time0 | 0.640516 | 0.014 | 0.018876 |
| 88 | Az_Glucose vs Cn_time0 | 0.714826 | 0.014 | 0.018876 |
| 89 | Az_Maltose vs Az_time0 | 0.354177 | 0.014 | 0.018876 |
| 90 | Az_Trehalose vs Cn_Serine | 0.303974 | 0.015 | 0.01978 |
| 91 | Az_Glucose vs Az_OCDCA | 0.524543 | 0.015 | 0.01978 |
| 92 | Az_none vs Az_Histidine | 0.304676 | 0.016 | 0.020645 |
| 93 | Cn_OCDCA vs Cn_Serine | 0.37752 | 0.016 | 0.020645 |
| 94 | Cn_none vs Az_OCDCA | 0.655985 | 0.017 | 0.021702 |
| 95 | Az_OCDCA vs Az_Serine | 0.451137 | 0.018 | 0.022737 |
| 96 | Az_OCDCA vs Cn_Histidine | 0.340439 | 0.02 | 0.025 |
| 97 | Cn_Trehalose vs Az_time0 | 0.253671 | 0.021 | 0.025979 |
| 98 | Az_Glucose vs Az_Histidine | 0.449534 | 0.024 | 0.029091 |
| 99 | Az_OCDCA vs Az_time0 | 0.237181 | 0.024 | 0.029091 |
| 100 | Cn_none vs Az_Histidine | 0.570105 | 0.026 | 0.030291 |
| 101 | Az_none vs Az_Serine | 0.353275 | 0.026 | 0.030291 |
| 102 | Az_Histidine vs Cn_Serine | 0.416123 | 0.026 | 0.030291 |
| 103 | Az_Histidine vs Az_Serine | 0.486974 | 0.026 | 0.030291 |
| 104 | Cn_none vs Az_Glucose | 0.468175 | 0.028 | 0.031698 |
| 105 | Cn_none vs Az_Serine | 0.504148 | 0.028 | 0.031698 |
| 106 | Az_none vs Cn_Histidine | 0.374464 | 0.028 | 0.031698 |
| 107 | Cn_none vs Cn_Serine | 0.387949 | 0.03 | 0.033333 |
| 108 | Az_Glucose vs Cn_Serine | 0.401983 | 0.03 | 0.033333 |
| 109 | Az_none vs Cn_Serine | 0.35481 | 0.031 | 0.034128 |
| 110 | Cn_none vs Az_none | 0.26749 | 0.035 | 0.038182 |
| 111 | Az_Glucose vs Cn_Histidine | 0.450071 | 0.041 | 0.044324 |
| 112 | Az_OCDCA vs Cn_Serine | 0.272749 | 0.044 | 0.047143 |
| 113 | Az_Glucose vs Az_Serine | 0.428448 | 0.05 | 0.053097 |
| 114 | Cn_none vs Cn_Histidine | 0.400049 | 0.053 | 0.055789 |
| 115 | Cn_Trehalose vs Cn_Serine | 0.281014 | 0.073 | 0.076174 |
| 116 | Cn_Serine vs Az_time0 | 0.214688 | 0.088 | 0.091034 |
| 117 | Cn_Histidine vs Az_Serine | 0.253594 | 0.099 | 0.101538 |
| 118 | Cn_Serine vs Az_Serine | 0.216278 | 0.13 | 0.132203 |
| 119 | Cn_Histidine vs Az_time0 | 0.246806 | 0.134 | 0.135126 |
| 120 | Cn_Histidine vs Cn_Serine | 0.143587 | 0.248 | 0.248 |
