# Supplementary material for: Modeling-Guided Amendments Lead to Enhanced Biodegradation in Soil
Source: mSystems. 2022 Aug 1;7(4):e00169-22. doi: 10.1128/msystems.00169-22 (PMC9426591; doi:10.1128/msystems.00169-22)
Supplement: FIG S2 [file msystems.00169-22-s0002.docx]

**Modelling-guided amendments lead to enhanced biodegradation in soil**

Kusum Dhakar^1,2¥^, Raphy Zarecki^1,2¥^, Shlomit Medina^1^, Hamam Ziadna^1^, Karam Igbaria^1^, Ran Lati^1^, Zeev Ronen^2 ϯ^, Hanan Eizenberg^1^ & Shiri Freilich^1^*^ϯ^

^1^Newe Ya'ar Research Center, Agricultural Research Organization, Ramat Yishay, Israel, ^2^Department of Environmental Hydrology & Microbiology, Zuckerberg Institute for Water Research, Jacob Blaustein Institutes for Desert Research, Ben-Gurion University of the Negev, Midreshet Ben-Gurion, 8499000, Israel,

^3^Albert Katz School for Desert Studies Jacob Blaustein Institutes for Desert Research, Ben-Gurion University of the Negev, Midreshet Ben-Gurion, 8499000, Israel,

^4^Junior Research Group Microbial Biotechnology, Leibniz Institute DSMZ, German Collection of Microorganisms and Cell Cultures, Braunschweig, Germany

^¥^equal contribution

^ϯ^ equal contribution

* Corresponding author (shiri@agri.gov.il,+972506220047)


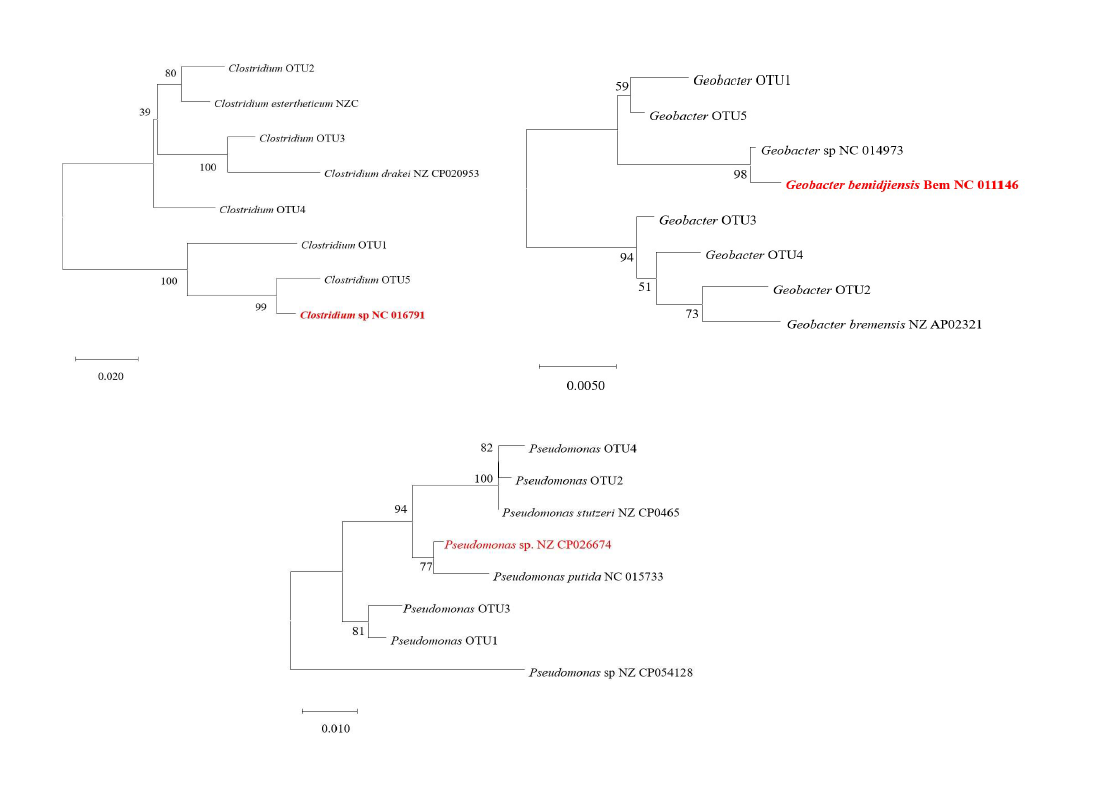


**Fig.S2.** Phylogenetic relationship of the most abundant OTUs of the corresponding genera and the top hit in BLAST. The tree was constructed in MEGA 7.0 based on the NJ method with bootstrap value 1000. Sequences with red bold are the selected genomes
