# Supplementary material for: Modeling-Guided Amendments Lead to Enhanced Biodegradation in Soil
Source: mSystems. 2022 Aug 1;7(4):e00169-22. doi: 10.1128/msystems.00169-22 (PMC9426591; doi:10.1128/msystems.00169-22)
Supplement: FIG S7 [file msystems.00169-22-s0007.docx]

**Modelling-guided amendments lead to enhanced biodegradation in soil**

Kusum Dhakar^1,2¥^, Raphy Zarecki^1,2¥^, Shlomit Medina^1^, Hamam Ziadna^1^, Karam Igbaria^1^, Ran Lati^1^, Zeev Ronen^2 ϯ^, Hanan Eizenberg^1^ & Shiri Freilich^1^*^ϯ^

^1^Newe Ya'ar Research Center, Agricultural Research Organization, Ramat Yishay, Israel, ^2^Department of Environmental Hydrology & Microbiology, Zuckerberg Institute for Water Research, Jacob Blaustein Institutes for Desert Research, Ben-Gurion University of the Negev, Midreshet Ben-Gurion, 8499000, Israel,

^3^Albert Katz School for Desert Studies Jacob Blaustein Institutes for Desert Research, Ben-Gurion University of the Negev, Midreshet Ben-Gurion, 8499000, Israel,

^4^Junior Research Group Microbial Biotechnology, Leibniz Institute DSMZ, German Collection of Microorganisms and Cell Cultures, Braunschweig, Germany

^¥^equal contribution

^ϯ^ equal contribution

* Corresponding author (shiri@agri.gov.il,+972506220047)


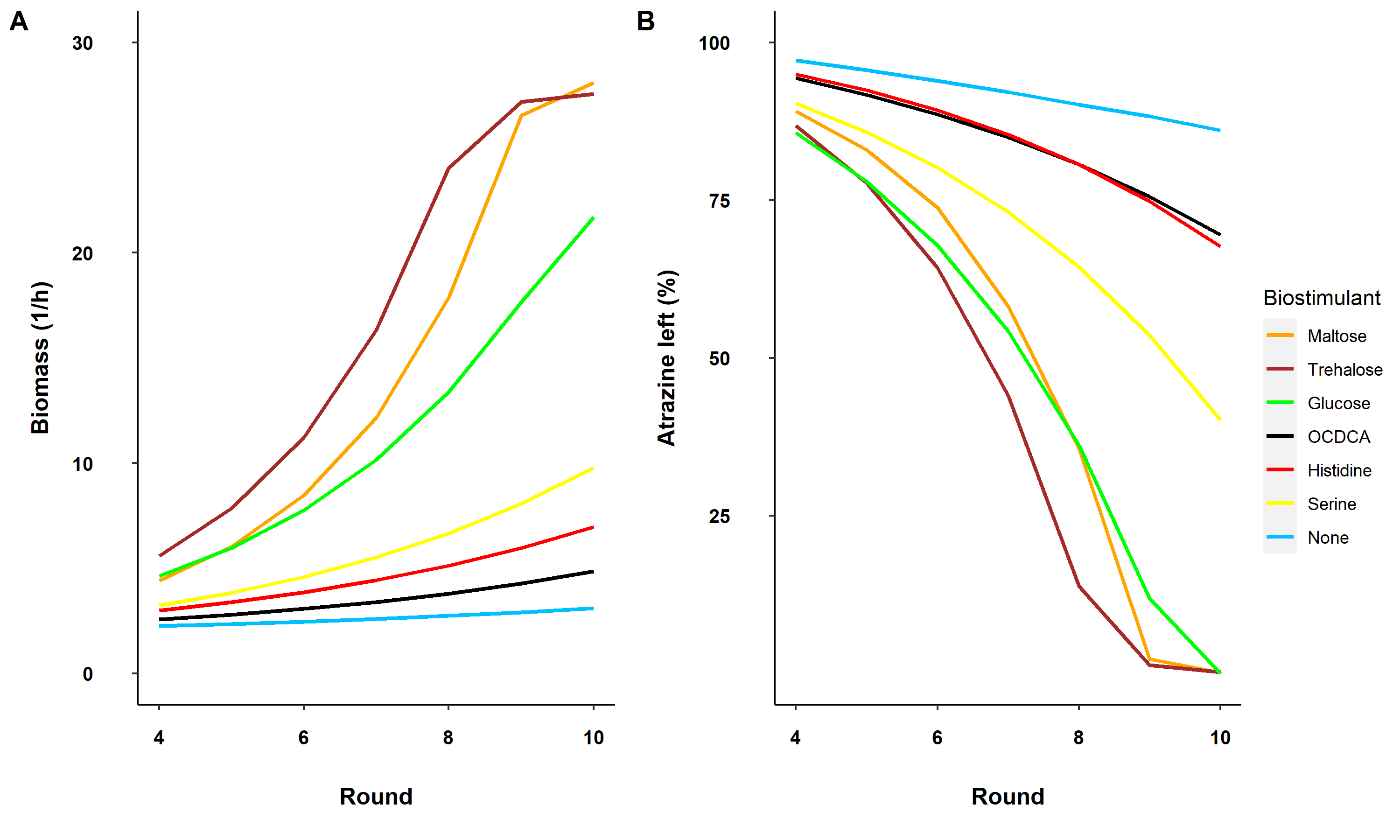


**Fig.S7.** Simulations for biomass generation (A) and atrazine degradation (B) by community (all four member species) under the influence of biostimulants
