# Supplementary material for: Modeling-Guided Amendments Lead to Enhanced Biodegradation in Soil
Source: mSystems. 2022 Aug 1;7(4):e00169-22. doi: 10.1128/msystems.00169-22 (PMC9426591; doi:10.1128/msystems.00169-22)
Supplement: TABLE S3 [file msystems.00169-22-s0010.docx]

**Modelling-guided amendments lead to enhanced biodegradation in soil**

Kusum Dhakar^1,2¥^, Raphy Zarecki^1,2¥^, Shlomit Medina^1^, Hamam Ziadna^1^, Karam Igbaria^1^, Ran Lati^1^, Zeev Ronen^2 ϯ^, Hanan Eizenberg^1^ & Shiri Freilich^1^*^ϯ^

^1^Newe Ya'ar Research Center, Agricultural Research Organization, Ramat Yishay, Israel, ^2^Department of Environmental Hydrology & Microbiology, Zuckerberg Institute for Water Research, Jacob Blaustein Institutes for Desert Research, Ben-Gurion University of the Negev, Midreshet Ben-Gurion, 8499000, Israel,

^3^Albert Katz School for Desert Studies Jacob Blaustein Institutes for Desert Research, Ben-Gurion University of the Negev, Midreshet Ben-Gurion, 8499000, Israel,

^4^Junior Research Group Microbial Biotechnology, Leibniz Institute DSMZ, German Collection of Microorganisms and Cell Cultures, Braunschweig, Germany

^¥^equal contribution

^ϯ^ equal contribution

* Corresponding author (shiri@agri.gov.il,+972506220047)

**Table S3.**

| 1. Predicted biomass of the individual microbial species in the model community supplemented with atrazine and biostimulants (one at a time) | | | | |
| --- | --- | --- | --- | --- |
| **Biostimulant** | ***Arthrobacter*** | ***Pseudomonas*** | ***Clostridium*** | ***Geobacter*** |
| Trehalose | 11.48419716 | 3.84455152 | 0.5 | 0.5 |
| Maltose | 10.65098251 | 0.5 | 0.5 | 0.5 |
| Glucose | 6.424593298 | 1.540009211 | 0.918963556 | 1.272465216 |
| OCDCA | 1.897009207 | 0.5 | 0.5 | 0.5 |
| Serine | 3.362584785 | 0.980066291 | 0.666905329 | 0.5 |
| Histidine | 2.23699503 | 1.192840548 | 0.5 | 0.5 |
| None | 1.086866975 | 0.5 | 0.5 | 0.5 |
|  |  |  |  |  |
| 1. Abundance of individual microbial group retrieved from the 16S rRNA amplicon gene sequencing from the atrazine treated groups supplemented with biostimulants | | | | |
| **Biostimulant** | ***Arthrobacter*** | ***Pseudomonas*** | ***Clostridium*** | ***Geobacter*** |
| D-Trehalose | 2.165476 | 1.232631 | 0.817588 | 0.505916 |
| D-Maltose | 1.965546 | 0.515979 | 0.817588 | 0.5 |
| D-Glucose | 1.424782 | 0.807787 | 0.649544 | 0.739839 |
| OCDCA | 1.582798 | 0.5 | 0.5 | 0.5 |
| L- Serine | 1.004723 | 0.637583 | 0.5 | 0.5 |
| L-Histidine | 0.5 | 1.060075 | 0.5 | 0.5 |
| None | 0.827228 | 0.5 | 0.5 | 0.5 |
